# Systematic analysis of protein–detergent complexes applying dynamic light scattering to optimize solutions for crystallization trials

**DOI:** 10.1107/S2053230X14027149

**Published:** 2015-01-01

**Authors:** Arne Meyer, Karsten Dierks, Rana Hussein, Karl Brillet, Hevila Brognaro, Christian Betzel

**Affiliations:** a Institute of Biochemistry and Molecular Biology, Laboratory for Structural Biology of Infection and Inflammation, University of Hamburg, c/o DESY, Building 22a, Notkestrasse 85, 22603 Hamburg, Germany; b XtalConcepts, Marlowring 19, 22525 Hamburg, Germany; c UMR 7242–IMPReSs Platform, ESBS, Pôle API, 300 Boulevard Sébastien Brant, CS10413, 67412 Illkirch CEDEX, France; d Multi User Center for Biomolecular Innovation, Department of Physics, São Paulo State University, UNESP/IBILCE, Caixa Postal 136, São José do Rio Preto-SP, 15054, Brazil

**Keywords:** dynamic light scattering, *n*-alkyl-β-d-maltopyranosides, micelle size, protein–detergent complex, hydrodynamic radius

## Abstract

Detergents are widely used for the isolation and solubilization of membrane proteins to support crystallization and structure determination. Detergents are amphiphilic molecules that form micelles once the characteristic critical micelle concentration (CMC) is achieved and can solubilize membrane proteins by the formation of micelles around them. The results are presented of a study of micelle formation observed by *in situ* dynamic light-scattering (DLS) analyses performed on selected detergent solutions using a newly designed advanced hardware device. DLS was initially applied *in situ* to detergent samples with a total volume of approximately 2 µl. When measured with DLS, pure detergents show a monodisperse radial distribution in water at concentrations exceeding the CMC. A series of all-*trans* *n*-alkyl-β-d-maltopyranosides, from *n*-hexyl to *n*-tetradecyl, were used in the investigations. The results obtained verify that the application of DLS *in situ* is capable of distinguishing differences in the hydrodynamic radii of micelles formed by detergents differing in length by only a single CH_2_ group in their aliphatic tails. Subsequently, DLS was applied to investigate the distribution of hydrodynamic radii of membrane proteins and selected water-insoluble proteins in presence of detergent micelles. The results confirm that stable protein–detergent complexes were prepared for (i) bacteriorhodopsin and (ii) FetA in complex with a ligand as examples of transmembrane proteins. A fusion of maltose-binding protein and the *Duck hepatitis B virus* X protein was added to this investigation as an example of a non-membrane-associated protein with low water solubility. The increased solubility of this protein in the presence of detergent could be monitored, as well as the progress of proteolytic cleavage to separate the fusion partners. This study demonstrates the potential of *in situ* DLS to optimize solutions of protein–detergent complexes for crystallization applications.

## Abstract 2

Application of *in situ* dynamic light scattering to solutions of protein–detergent complexes permits characterization of these complexes in samples as small as 2 µl in volume.

## 1. Introduction   

Detergents are bifunctional molecules with amphipathic properties. Because of these amphipathic properties, pure detergent monomers show peculiar solubility properties in water such that once a minimum concentration (the critical micelle concentration or CMC; Birdi, 1997 ▶) is achieved, all further additions of detergent molecules go into the formation of micelles. Micelles are soluble assemblies of detergent molecules that in water shield their hydrophobic tails within a layer of hydrophilic head groups exposed to the solution. Detergents in solution exist in monomer–micelle equilibria, and the amount of free monomers in solution remains constant at detergent concentrations higher than the CMC (Dominguez *et al.*, 1997 ▶). The CMC can be determined by dynamic light scattering (Vulliez-Le Normand & Eiselé, 1993 ▶); the scattered light intensity increases with the detergent concentration. In general, a detergent must be used at concentrations above its CMC in order to act as an effective solubilizing agent of hydrophobic molecules such as membrane proteins (Arnold & Linke, 2008 ▶). For structure analysis of membrane proteins, the most common use of detergents is to keep a membrane protein in a functional, correctly folded state in the absence of the biological membrane (Privé, 2007 ▶; Seddon *et al.*, 2004 ▶) both in solution and in the crystal. A significant obstacle in membrane-protein research and, in particular, in membrane-protein crystallization is the need to identify a suitable detergent and concentration that, in combination with buffer and additives, will maintain the stability and biological functionality of a given protein as a protein–detergent complex (PDC) during solubilization and crystallization (Oliver *et al.*, 2013 ▶). The experiments described here show that dynamic light scattering is a non-invasive method that can be used routinely to identify and optimize stable, soluble PDCs of proteins prior to crystallization experiments.

By forming micelles, detergents provide an amphipathic environment for the hydrophobic regions of membrane proteins. It is expected that intact detergent-solubilized membrane proteins will appear as PDCs with slightly but detectably different radii to those of pure micelles and, more importantly, they will show a dominant single-peak radial distribution when measured using DLS. On the other hand, poorly soluble or insoluble proteins can present a complex radial distribution with much larger radii, which is most likely to be a result of amorphous aggregation resulting from denatured or misfolded proteins (total or partial) or incomplete coverage of hydrophobic regions of the protein. For all experiments, the recently developed advanced DLS instrument SpectroLight 600 (Xtal­Concepts GmbH, Hamburg, Germany) was used. This instrument is capable of performing *in situ* DLS measurements in individual droplets in a tray without having to open any of the individual wells. It is designed to measure trays in an automated fashion, allowing the usage of standard crystallization plates for serial investigations. PDCs were found to be slightly, but measurably, different in size compared with uncomplexed micelles in solution. The results indicate that DLS is indeed a suitable diagnostic technique for the identification and characterization of PDCs. Furthermore, a poorly soluble or insoluble protein mixture can easily be identified by DLS, as can be failures to achieve a PDC, whether owing to the presence of a misfolded protein or an unsuitable detergent.

## 2. Materials and methods   

Serial *in situ* DLS analyses were carried out using a newly developed instrument, the SpectroLight 600 (XtalConcepts GmbH, Hamburg, Germany). Samples were pipetted onto a 72-well Terasaki plate (Nunclon Delta; catalogue No. 1-36528, Nunc GmbH, Wiesbaden, Germany) in volumes of ∼2 µl. Prior to use, the plates were filled with paraffin oil (paraffin oil light; catalogue No. A4692, AppliChem, Darmstadt, Germany) to protect the sample solutions from drying out. The laser wavelength used was 660 nm at a power of 100 mW. The scattering angle for placement of the detector was fixed at 150°. All investigated sample solutions were aqueous; therefore, the refractive index of water (1.33) was used for all calculations. All samples were measured at 293 K.

### 2.1. *n*-Alkyl-maltopyranosides   

The *n*-alkyl-β-d-maltopyranosides used in these experiments were the following: *n*-hexyl-β-d-maltopyranoside (catalogue No. A6820,0001, AppliChem, Darmstadt, Germany), *n*-octyl-β-d-maltopyranoside (catalogue No. A6809,0001, AppliChem), *n*-nonyl-β-d-maltopyranoside (catalogue No. 59965-1G, Sigma–Aldrich, Hamburg, Germany), *n*-decyl-β-d-maltopyranoside (catalogue No. D7658, Sigma–Aldrich), *n*-undecyl-β-d-malto­pyranoside (catalogue No. 94206, Sigma–Aldrich), *n*-dodecyl-β-d-maltopyranoside (catalogue No. D4641, Sigma–Aldrich), *n*-tridecyl-β-d-maltopyranoside (catalogue No. 16321, Sigma–Aldrich) and *n*-tetradecyl-β-d-maltopyranoside (catalogue No. A4810,0250, AppliChem).

All detergents were obtained commercially. To prepare sample solutions, solid *n*-alkyl-β-d-maltopyranosides were dissolved in pure water exceeding the CMC by three to ten times (Table 1 ▶) at an ambient temperature of 293 K. Prior to DLS analysis all samples were centrifuged at 16 100*g* for 60 min (Centrifuge 5415 R, Eppendorf, Hamburg, Germany) and filtered through a 0.2 µm filter (Ultrafree-MC Centrifugal filter devices, 0.5 ml; catalogue No. PR02905, Millipore, Schwalbach, Germany). DLS measurements were performed in replicates of 25 (30 s data-recording time) for each sample investigated and standard errors were estimated from the scatter of the replicates.

### 2.2. Bacteriorhodopsin   

Bacteriorhodopsin (BR) from *Halobacterium salinarum* is a transmembrane protein with a seven-α-helical domain and a molecular mass of 27 kDa (Subramaniam & Henderson, 2000 ▶). It acts as a photon-driven proton pump (Miercke *et al.*, 1989 ▶). Bacteriorhodopsin was purchased from Sigma–Aldrich as purple membrane (catalogue No. B0184), and a protein–detergent complex was prepared by a modification of the protocol originally published by Miercke *et al.* (1989 ▶). By applying the low sample volume, *in situ* capabilities of DLS technology, a more efficient purification protocol could be established to separate BR from the purple membrane, thereby avoiding the chromatography steps and the need for another detergent as a prelude to CHAPSO. This purification protocol requires six steps, each of which was a buffer exchange performed in a 0.5 ml concentrator tube (catalogue No. UFC500308, Millipore, Schwalbach, Germany). The six steps are as follows. Step 1: BR-containing purple membrane was suspended in 500 µl 16 m*M* CHAPSO (catalogue No. C4695, Sigma–Aldrich), 100 m*M* sodium chloride (catalogue No. 1064045000, Merck, Darmstadt, Germany) and 20 m*M* sodium acetate (catalogue No. 106282500, Merck) pH 5.0, transferred to a concentrator tube and concentrated to 20 mg ml^−1^. Steps 2 and 3: the first buffer was exchanged twice by the addition of 16 m*M* CHAPSO, 100 m*M* NaCl and 0.2% Triton X-100 in 20 m*M* sodium acetate pH 5.0 in a 1:1 ratio and concentrated again to 20 mg ml^−1^ by centrifugation for 30 min at 800*g*. Step 4: BR was diluted to 2 mg ml^−1^ by adding 2% Triton X-100 in 20 m*M* sodium acetate to the concentrator and concentrated to 4 mg ml^−1^. Step 5: BR was diluted again to 2 mg ml^−1^ by the addition of 2% Triton X-100 in 20 m*M* sodium acetate to the concentrator and concentrated to 4 mg ml^−1^. Step 6: BR was finally diluted to 2 mg ml^−1^ in 2% Triton X-100 in 20 m*M* sodium acetate and centrifuged for 20 min at 16 000*g*. The BR solution was analyzed *via in situ* DLS after each step (Fig. 2 and Supplementary Figs. S2*a*–S2*f*, S3 and S4). The results were interpreted as a successful isolation of BR from the purple membrane at a concentration of 2 mg ml^−1^, probably as a BR–Triton X-100 complex with a hydrodynamic radius of 5.6 nm (Table 2 ▶).

### 2.3. Maltose-binding protein–*Duck hepatitis B virus* X fusion protein   

The dHBx protein of *Duck hepatitis B virus* (DHBV; de Moura *et al.*, 2005 ▶) was purified following a published purification protocol (Liu *et al.*, 2009 ▶). DHBx has a molecular weight of 14 kDa, corresponding to 114 amino acids. It is considered to be a multifunctional regulator (Tang *et al.*, 2008 ▶). The maltose-binding protein (Mbp) from *Escherichia coli* is periplasmatic protein from the maltose transport machinery, that belongs to the periplasmic permease family (Bassford, 1990 ▶) with a molecular weight of 42 kDa. The combination of Mbp with dHBx was intended to overcome the low water solubility of dHBx in order to avoid the formation of insoluble aggregates known as inclusion bodies (Lilie *et al.*, 1998 ▶; Kapust & Waugh, 1999 ▶; Sachdev & Chirgwin, 1998 ▶). The Mbp can be released from the Mbp-dHBx fusion protein by *Tobacco etch virus* endopeptidase (TEV), a sequence-specific cysteine protease that cleaves a linker region between the two proteins (Liu *et al.*, 2009 ▶). Mbp-dHBx forms a soluble oligomer in aqueous solution and even proteolytic cleavage by TEV has no effect on the hydrodynamic radius, as indicated by *in situ* DLS. To dissolve the Mbp-dHBx oligomer, tridecyl-β-d-maltopyranoside (TDM) was used to form the PDC and also to separate Mbp from dHBx after cleavage by TEV.

### 2.4. Enantiopyochelin receptor FetA   

The FetA protein is a 81 kDa integral membrane protein inserted as a β-barrel into the outer membrane of the Gram-negative bacterium *Pseudomonas fluorescens* (Brillet *et al.*, 2011 ▶). FetA specifically transports the siderophore enantiopyochelin (EPch), the enantiomer of pyochelin (Pch) produced by *P. aeruginosa* (Youard *et al.*, 2007 ▶; Cobessi *et al.*, 2005 ▶). After iron chelation in the extracellular medium Pch-Fe and EPch-Fe are recognized and transported by FptA and FetA, respectively (Schalk *et al.*, 2012 ▶). FetA is involved in iron uptake as a TonB-dependent transporter (TBDT; Schalk *et al.*, 2012 ▶; Yue *et al.*, 2003 ▶). After iron chelation in the extracellular medium Pch-Fe and EPch-Fe are recognized and transported by their specific TBDT based on the configuration of the C4′′ and C2′′ chiral centres of the siderophore (Brillet *et al.*, 2011 ▶). FetA was isolated and purified according to Brillet *et al.* (2011 ▶). The FetA–Epch-Fe complex was used at a concentration of 5 mg ml^−1^ in 10 m*M* Tris pH 8.0 and in the presence of 0.75%(*m*/*v*) *n*-octylpentaoxyethylene (C_8_E_5_; Bachem, Bubendorf, Switzerland). The FetA–EPch-Fe complex was investigated by *in situ* DLS prior to and after the addition of TDM (tridecyl-β-d-maltopyranoside) at various concentration ratios. The rationale behind the addition of TDM to the sample was to increase the solubility of FetA–EPch-Fe by forming a protein–detergent complex. The 13-carbon aliphatic tail of TDM has more hydrophobic potential than C_8_E_5_ with only eight aliphatic C atoms. By covering the hydrophobic moieties stabilizing the aggregation in aqueous solution, a PDC could be obtained.

## 3. Results and discussion   

### 3.1. *n*-Alkyl-maltopyranosides   

**Figure 1:**
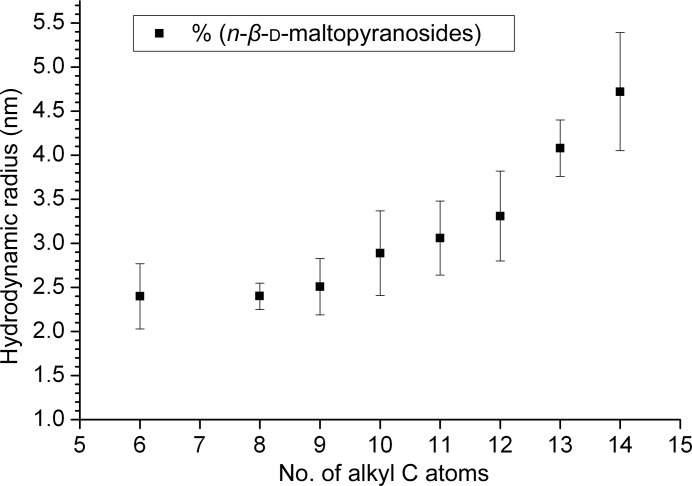
Correlation plot of hydrodynamic micelle radii of the *n*-alkyl-d-maltopyranosides in water against *n*-alkyl chain length.

Most micelles show a monodisperse or unimodal radial distribution when analyzed by DLS. Histograms corresponding to individual DLS measurement series are summarized in Supplementary Fig. S1. Columns (red blocks) in the histograms represent the relative concentrations of particles of specific radii independent of their scattered light intensity. Intensities of light scattered by particles are also indicated (blue curves) in arbitrary units. The radial distribution plots indicate the radius on the vertical axis *versus* time on the horizontal axis. Results and radial distributions correspond well to previously published data based on conventional DLS measurements in cuvettes (Vulliez-Le Normand & Eiselé, 1993 ▶). A summary of *in situ* DLS analyses for the selected *n*-alkylmaltopyranosides is displayed in Table 1 ▶ and shown in Fig. 1 ▶. All maltopyranosides with alkyl-chain lengths from six to 14 alkyl C atoms (except for *n*-heptyl-β-d-maltopyranoside) were investigated at concentrations above the CMC. The corresponding *n*-alkylmaltoside micelles show peak radial values *R*_h_ of between 2.4 ± 0.4 and 4.7 ± 0.7 nm (Table 1 ▶). These values correspond approximately to the long half-axis values derived from small-angle X-ray scattering (SAXS) experiments on these micelles. Interpretation of the SAXS data indicates oblate-shaped models. For octyl-β-d-maltopyranoside (OM), the long half axis has been measured by SAXS (Oliver *et al.*, 2013 ▶) to be 1.8–1.9 nm, for dodecyl-β-d-maltopyranoside (DDM) SAXS revealed the long half axis to be 2.8 nm and *in situ* DLS measurements showed an *R*_h_ of 3.3 ± 0.5 nm. The absolute size values measured by DLS and SAXS differ only slightly and are probably owing to minor uncertainties in the viscosity parameters. The plot in Fig. 1 ▶ shows the *R*_h_ values as a function of alkyl-chain length. The data suggest that a difference of a single CH_2_ group can be detected by *in situ* DLS throughout the range of hydrodynamic radii measured. The average *R*_h_ increases by about 0.4 Å per single CH_2_ group added to the alkyl chain (Fig. 1 ▶). Note, however, that the alkyl-length-dependent micelle-size enlargement is not linear.

### 3.2. Bacteriorhodopsin   

**Figure 2:**
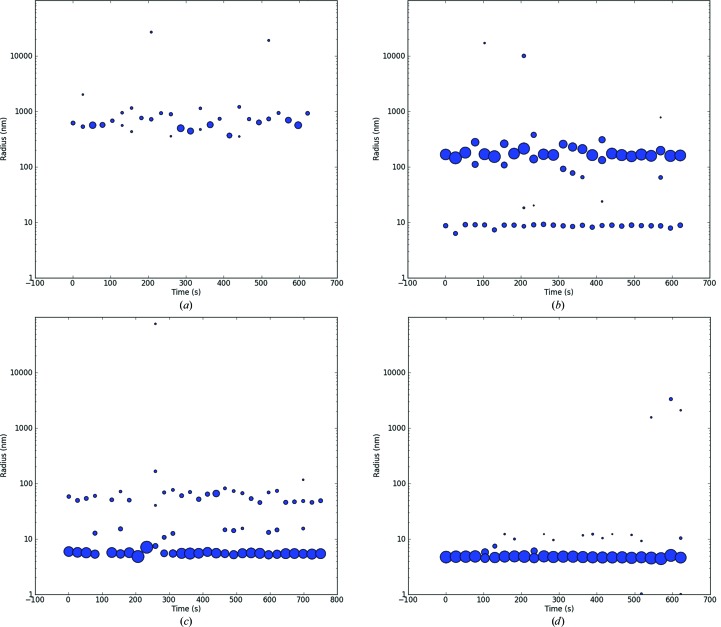
Radial distribution plots of the bacteriorhodopsin/purple membrane assembly (*a*), the intermediate state during the isolation of the protein from the membrane (*b*) and the assumed PDC of bacteriorhodopsin (*c*). (*a*) Radial distribution of bacteriorhodopsin/purple membrane assembly (BR) at a concentration of 20 mg ml^−1^ in 16 m*M* CHAPSO, 100 m*M* sodium chloride, 20 m*M* sodium acetate pH 5.0. (*b*) Radial distribution of BR at 20 mg ml^−1^ in 16 m*M* CHAPSO, 100 m*M* NaCl, 20 m*M* sodium acetate pH 5.0 after the addition of 2%(*v*/*v*) Triton X-100 in 20 m*M* sodium acetate as the first step of buffer exchange *via* a concentrator tube. The sample was reconcentrated for 30 min at 800*g* and then analyzed by *in situ* DLS. (*c*) The radial distribution of BR at 2.0 mg ml^−1^ after complete buffer exchange to 2%(*v*/*v*) Triton X-100 in 20 m*M* sodium acetate, centrifugation for 20 min and finally centrifugation at 16 000*g* in an Eppendorf tube prior to *in situ* DLS analysis. (*d*) Radial distribution of pure Triton-X micelles at a 2%(*v*/*v*) concentration in 20 m*M* sodium acetate as a control. The spot diameter represents the relative scattered light intensity of the detected particles in arbitrary units.

BR was isolated from the colloidally suspended purple membrane (PM) of *H. salinarum* by the replacement of CHAPSO by successive addition of 2%(*v*/*v*) Triton X-100. All steps were carried out using a concentrator tube. The colloidally suspended PM and the BR solutions of the intermediate steps were analyzed by DLS (Fig. 2 ▶). The PM colloids could be measured as 600 nm (Fig. 2 ▶ *a*). During the isolation of BR from the PM, the intermediate states were analyzed by *in situ* DLS. Two fractions, with particle radii of ∼200 and ∼9 nm, were found. The 9 nm fraction was interpreted as a precursor of the BR–detergent complex (Fig. 2 ▶ *b*). After completion of the process, the isolated particle fraction has an *R*_h_ of 5.6 ± 0.5 nm and is assumed to be the BR PDC (Fig. 2 ▶ *c*). This was later confirmed by SDS–PAGE (Supplementary Fig. S3, lanes 7 and 8). The BR PDC was slightly larger compared with the *R*_h_ obtained for pure Triton X-100 micelles, which showed an *R*_h_ of 4.9 ± 0.6 nm (Fig. 2 ▶ *d*).

### 3.3. Maltose-binding protein–*Duck hepatitis B virus* X fusion protein   

**Figure 3:**
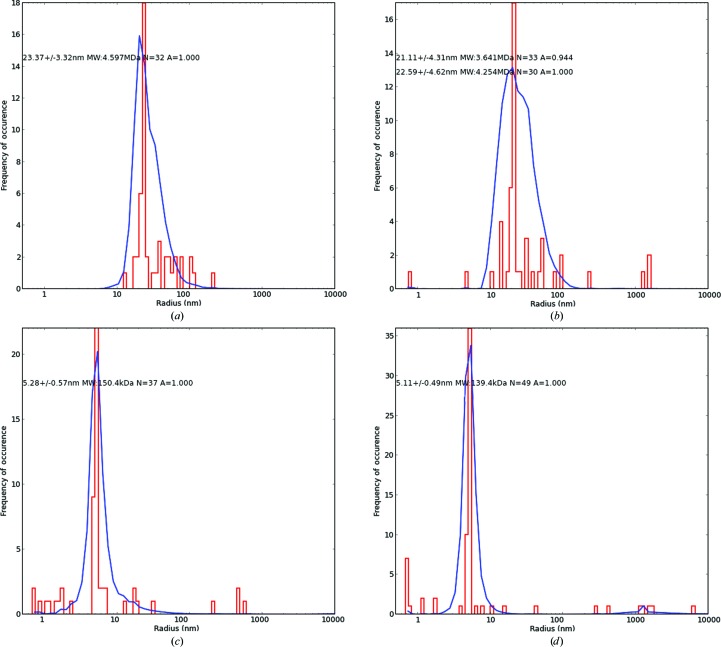
Radial distribution histograms characterizing the Mbp-dHBx fusion using *in situ* DLS analysis. (*a*) Mbp-dHBx at 2.0 mg ml^−1^ in 20 m*M* Tris–HCl, 50 m*M* NaCl, 1 m*M* DTT, 1 m*M* EDTA pH 7.4. (*b*) Mbp-dHBx (2.0 mg ml^−1^) in 20 m*M* Tris–HCl, 50 m*M* NaCl, 1 m*M* DTT, 1 m*M* EDTA pH 7.4, 6.3 m*M* TDM in the presence of the protease TEV. (*c*) Mbp-dHBx (2.0 mg ml^−1^) in 20 m*M* Tris–HCl, 50 m*M* NaCl, 1 m*M* DTT, 1 m*M* EDTA pH 7.4 and 9.8 m*M* TDM in the absence of TEV. (*d*) Control: pure 19 m*M* TDM.

The DLS histogram of Mbp-dHBx (Fig. 3 ▶ *a*) shows radial distributions of the Mbp-dHBx fusion protein dissolved in a buffer consisting of 0.05% CHAPS, 50 m*M* NaCl, 20 m*M* Tris, 1 m*M* EDTA, 1 m*M* DDT pH 7.4. Under these buffer conditions Mbp-dHBx shows an *R*_h_ of 23.4 nm, which corresponds to a protein oligomer. In contrast, the expected *R*_h_ for a 42 kDa fusion protein in monomeric form would be around 3–4 nm. In the presence of TDM at a concentration of 6.3 m*M* and in the presence of the protease TEV at a molar ratio of 1:100, DLS gives a species with a hydrodynamic radius of ∼22 nm (Fig. 3 ▶ *b*), suggesting that the oligomer is still present. Hence, neither TDM at this concentration nor TEV have a dissolving effect on the oligomer. However, when TDM was added to a concentration of 9.8 m*M* in the absence of TEV, the 22–23 nm oligomer could no longer be detected by *in situ* DLS. Instead of the 22–23 nm oligomer, an *R*_h_ of 5.3 ± 0.6 nm (Fig. 3 ▶ *c*) corresponding to the fusion protein was detected. Both concentrations (6.3 and 9.8 m*M*) are greater than the CMC for TDM (0.033 m*M*). However, TDM at 6.3 m*M* is apparently insufficient to dissolve the Mbp-dHBx aggregate. Obviously, the presence of the detergent in the form of micelles is not sufficient to induce decomposition of the Mbp-dHBx aggregate. Instead, the TDM:protein ratio seems to the crucial factor. TDM at 9.8 m*M* is sufficient to dissolve the aggregates. One explanation may be that interactions of the detergent aliphatic tail with surface-located hydrophobic amino acids destabilize the aggregate by weakening van der Waals interactions. It is probable that micelle-forming detergent molecules and detergent molecules interacting with the protein exist in equilibrium conditions. Thus, micelles have to exceed a certain concentration to provide sufficient detergent to destabilize protein aggregates and stabilize protein–detergent complexes. As a first control, pure TDM micelles have been analyzed and show an *R*_h_ of 5.1 ± 0.5 nm (Fig. 3 ▶ *d*). In order to exclude protein-unfolding effects induced by TDM, the ability to bind to immobilized maltose on affinity gel chromatography was assumed to indicate correctly folded Mbp (and most probably also dHBx). A completely or partially denaturated protein would have lost its maltose specificity (Dhuna *et al.*, 2005 ▶). SDS–PAGE of the protein (Supplementary Fig. S7, lanes 1, 2 and 3) shows that a significant amount of Mbp-dHBx was eluted from the column with maltose in the mobile phase, indicating that the protein was still in a correctly folded state.

**Figure 4:**
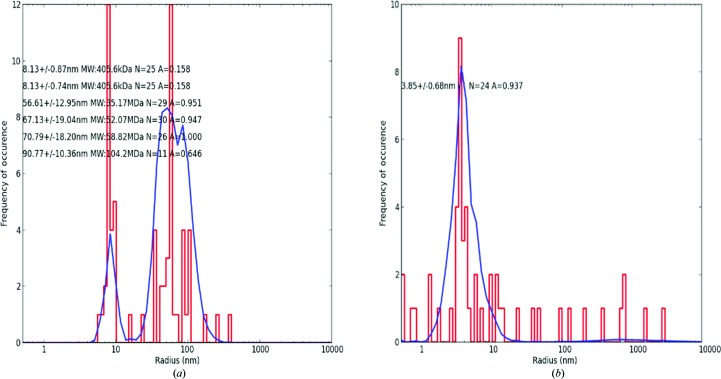
DLS histograms of the Mbp-dBHx oligomer in the presence of 9.8 m*M* TDM and after addition of the TEV protease in a 1:100 ratio. (*a*) 4 min after TEV addition, (*b*) 4 h after TEV addition.

Successful cleavage of Mbp-dHBx by TEV and separation of the products Mbp and dHBx could be monitored by *in situ* DLS when 9.8 TDM and 1:100 TEV were added (Fig. 4 ▶). 4 min after the addition, two predominant peaks, one with an *R*_h_ of 8.13 ± 0.74 nm and one with a broader *R*_h_ of 57–67 nm, were observed (Fig. 4 ▶ *a*), which could be interpreted as an intermediate state of the cleavage process. 4 h after TEV addition a broad predominant peak with a radius of 3.85 ± 0.68 nm was present, corresponding to Mbp and dHBx and also the fusion protein. Partial cleavage of Mbp-dHB in the presence and also in the absence of TDM was confirmed by SDS–PAGE (Supplementary Fig. S5, lane 4). Cleavage products and remaining fusion proteins correspond to the 14 kDa (dHBx), 42 kDa (Mbp) and ∼56 kDa (Mbp-dHBx) bands.

### 3.4. FetA–Epch-Fe complex   

**Figure 5:**
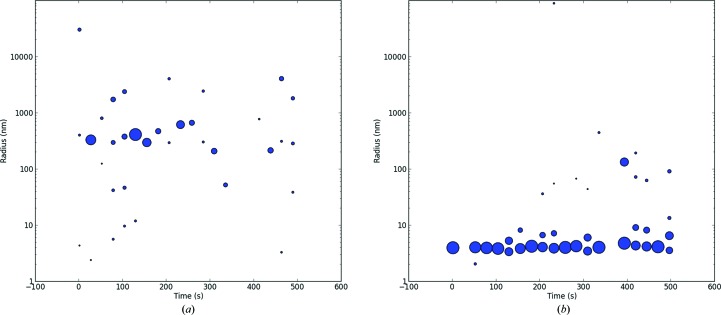
Radial distribution of FetA in complex with EPch-Fe investigated by *in situ* DLS. (*a*) FetA–EPch-Fe at 5 mg ml^−1^ in 10 m*M* Tris pH 8.0, 0.75%(*m*/*v*) *n*-octylpentaoxyethylene. (*b*) FetA–EPch-Fe at 5 mg ml^−1^ in 10 m*M* Tris pH 8.0, 0.75%(*m*/*v*) *n*-octylpentaoxyethylene + 9.5 m*M* TDM.

A 2 µl aliquot of the FetA–Epch-Fe complex at 5 mg ml^−1^ in 10 m*M* Tris pH 8.0, 0.75%(*m*/*v*) *n*-octylpentaoxyethylene was used for *in situ* DLS measurements. In the absence of TDM, the FetA sample shows a polydisperse radial distribution, indicating a highly aggregated protein with a predominant peak at ∼300 nm (Fig. 5 ▶ *a*). Analogous to Mbp-dHBx (§[Sec sec3.3]3.3), addition of TDM caused a significant change in the radial distribution. The solution became monodisperse after a few minutes and the 300 nm radius corresponding to an aggregate disappeared, while a 3.5 nm particle radius appeared (Fig. 5 ▶ *b*). The 13 C-atom aliphatic tail of TDM appears to reduce the number of hydrophobic interactions and, in analogy to Mbp-dHBx, supports the formation of a monodisperse and water-soluble PDC. Remarkably, the PDC of FetA has an *R*_h_ of 3.8 nm, which is smaller than the micelles of the pure detergent TDM (4.11 nm; Table 1 ▶). This suggests that a PDC is not a ‘loaded’ micelle but a complex of the protein in a certain stoichiometric ratio with the detergent that might be larger or smaller than the micelles of the pure detergent.

## 4. Conclusion   

DLS has already been shown to be an appropriate technology for interrogating detergent micelles and PDCs in solution. The ability to use this technique *in situ* on (relatively) small droplets shows that it may be a new, and perhaps a highly appropriate, technology for analyzing, measuring and scoring radial distributions of detergent–protein solutions, and particularly for the identification and characterization of PDCs. This technology allows the optimization of solutions for crystallization experiments prior to crystallization screening experiments using as criteria (i) the absence of large oligomers or aggregates and (ii) the presence of monodisperse solutions with radii corresponding to monomeric PDCs.

*In situ* DLS systems allow the detection of even small differences in the hydrodynamic radii of pure detergent micelles and the corresponding protein–detergent mixture. These differences certainly depend on a variety of variables such as the dimensions of the protein, the mode and strength of protein–detergent interaction and possibly the ratio of surface-exposed hydrophobic and hydrophilic moieties. It is also shown that the molecular weight of the protein does not necessarily correlate with its measured PDC dimensions (Table 2 ▶), suggesting that the assumption that PDCs are mainly spherical particles is probably overly simple. However, for successful crystallization of membrane proteins and other water-insoluble proteins, appropriate PDC formation is important, in addition to other criteria (Zhang *et al.*, 2003 ▶). The procedures and technology introduced here will certainly support the preparation of monodisperse PDC solutions and, we hope, increase the success rate of the production of membrane-protein crystals that are suitable for diffraction.

## Figures and Tables

**Table 1:** Hydrodynamic radii of micelles formed by *n*-alkyl--D-maltopyranosides

No. of *n*-alkyl C atoms	CMC (m*M* in H_2_O)/ concentration used (m*M*)	Mean *R*_h_ of micelles (nm)
6	210† (8.9%)/710.6	2.4 0.4
8	19.5‡ (0.89%)/114.4	2.4 0.2
9	6† (0.28%)/60	2.5 0.3
10	1.8† (0.087%)/18	2.9 0.5
11	0.59† (0.029%)/5.59	3.1 0.4
12	0.17† (0.00887%)/1.17	3.3 0.5
13	0.033† (0.0017%)/99.1	4.1 0.3
14	0.01† (0.00054%)/30.48	4.7 0.7

† Affimetrix (http://www.affymetrix.com).

‡ SigmaAldrich (http://www.sigmaaldrich.com/catalog/product/sigma/19181).

**Table 2:** Summary of sizes and dimensions of PDCs and micelles The volumes are calculated assuming that the PDCs and micelles are spherical and hydrated particles. Remarkable differences in the PDC loading could be observed under the simplified assumption that, for example, BR occupies 30.2nm^3^ per kDa. In contrast, FetA has a volume of only 2.99nm^3^ per kDa.

Protein	Molecular weight (kDa)	Protein concentration (mgml^1^)	PDC *R*_h_ (nm)	PDC spherical volume (nm^3^)	Micelles *R*_h_ (nm)	Micelles spherical volume (nm^3^)	PDC nm^3^ per kDa
BR	27	2.0	5.61 0.51	817.28	4.90 0.59	407.73	30.23
Mbp-dHBx	56	2.0	5.28 0.57	616.58	5.11 0.49	558.92	11.01
FetA	77	5.0	3.80 0.28	229.85	5.11 0.49	558.92	2.99
